# Preliminary assessment of age- and sex-related differences in brain volume using Quantib™ brain quantification: a study in a Vietnamese cohort

**DOI:** 10.3389/fneur.2025.1552559

**Published:** 2025-08-22

**Authors:** Dang Luu Vu, Thi Phuong Lien Do, Khoi Viet Nguyen, Huu An Nguyen, Quang Anh Nguyen, Van Khang Le, Ngoc Trang Nguyen, Hong Khoi Vo, Van Hoang Nguyen, Cong Tien Nguyen, Laurent Pierot

**Affiliations:** ^1^Radiology Center, Bach Mai Hospital, Hanoi, Vietnam; ^2^Department of Radiology, Hanoi Medical University, Hanoi, Vietnam; ^3^Department of Radiology, Dong Da General Hospital, Hanoi, Vietnam; ^4^Department of Neuroradiology, Hôpital Maison-Blanche, Université Reims-Champagne-Ardenne, Reims, France; ^5^Department of Neuro Intensive Care and Emergency Neurology, Neurology Center, Bach Mai Hospital, Hanoi, Vietnam; ^6^Department of Neurology, Hanoi Medical University, Hanoi, Vietnam; ^7^Department of Neurology, Faculty of Medicine, VNU University of Medicine and Pharmacy, Vietnam National University, Hanoi, Vietnam

**Keywords:** brain volume assessment, high-field MRI, Quantib™ brain software, cognitive health, age-related differences, sex-related differences, Vietnamese population

## Abstract

**Objective:**

This study evaluates age- and sex-related differences in brain volume, including normalized gray matter (nGM), normalized white matter (nWM), cerebrospinal fluid (CSF) volume, and total intracranial volume (TIV) in cognitively normal adults using automatic volume segmentation on 3.0 Tesla MRI.

**Methods:**

A prospective cross-sectional study conducted from October 2021 to September 2022 included 110 cognitively normal participants. They were divided into younger (18–35 years) and older (60–80 years) groups. Brain MRI were performed at Bach Mai Hospital, and volumetric analysis utilized automated segmentation software (Quantib™ Brain, GE Healthcare). Differences in brain volumes were analyzed by age and sex.

**Results:**

The younger group comprised 57 participants (30 females, 27 males; mean age 28), and the older group comprised 51 participants (32 females, 19 males; mean age 66). nGM was significantly higher in younger than older groups (*p* < 0.001), with no significant sex differences (*p* = 0.51). nGM showed an inverse correlation with age in younger males (r = −0.56, *p* < 0.001) and older males (r = −0.52; *p* = 0.02), but not in females (*p* = 0.77 in younger group and *p* = 0.07 in older group). nWM was also higher in younger groups (*p* = 0.02), with no significant sex differences (*p* = 0.10) or correlation with age across all groups (*p* > 0.05). CSF volume was significantly higher in males (*p* = 0.001) and older groups (*p* < 0.001). A positive correlation was noted between CSF volume and age in younger males (r = 0.41; *p* = 0.02), but not in other groups. TIV was higher in males (*p* < 0.01) and in younger groups (*p* < 0.001), with no correlation with age in any group (*p* > 0.05).

**Conclusion:**

This preliminary study suggests potential age- and sex-related differences in brain volume indices among cognitively normal Vietnamese adults. Additional studies with larger and more representative populations are warranted to confirm and expand upon these findings.

## 1 Introduction

Structural brain changes associated with age and sex in adults across different racial and ethnic groups remain poorly understood. Early investigations into brain volume relied on autopsy studies with limited sample sizes, such as those measuring brain weight in 1978 (1). The advent of neuroimaging technologies, starting with the introduction of the CT scanner in 1971 (2) and later magnetic resonance imaging (MRI) in 1977 (3), marked a significant breakthrough in the study of living human brain structures. These advances have since enabled larger cohort studies, offering deeper insights into brain structure and function (4).

Brain volume, encompassing gray matter (GM), white matter (WM), cerebrospinal fluid (CSF), and total intracranial volume (TIV), undergoes dynamic changes influenced by aging and sex (5, 6). GM volume generally declines linearly with age due to synaptic loss, dendritic shrinkage, and neuronal apoptosis, whereas WM follows a nonlinear trajectory-typically increasing into middle age due to ongoing myelination and then declining due to demyelination and vascular changes (4, 7–11). These volumetric shifts serve as critical biomarkers for understanding neurodevelopment, aging processes, and the progression of neurological disorders (12, 13).

Sex-related differences have also been consistently reported, with males typically showing higher absolute GM and WM volumes, although normalization for TIV often attenuates these differences (14–16). Moreover, the rates of age-related brain volume decline may vary between men and women, potentially influenced by hormonal and genetic factors (4, 17, 18). While numerous studies have explored age- and sex-related differences in brain volume across various populations, these investigations are predominantly based on Western cohorts (13, 19–22). Recent evidences have suggested that brain morphology can differ significantly across populations due to ethnicity and genetic background (12, 23, 24).

With technological advances not only in high-field MRI (25, 26) but also in brain segmentation techniques - from manual to semi-automatic and fully automated methods such as Quantib™ Brain (GE Healthcare) (27–29)—the assessment of human brain volume has become increasingly feasible. Despite these developments, research involving Mainland Southeast Asian cohorts—specifically Vietnamese individuals—remains scarce (12, 13, 19–24). Given that age- and sex-related volumetric patterns may vary across ethnic groups, population-specific normative values are essential for improving the diagnostic accuracy of neurological disorders. In this context, the present study aims to provide preliminary data on age- and sex-related brain volume differences in cognitively normal Vietnamese adults, utilizing Quantib™ Brain on 3.0-Tesla MRI data. We hypothesize that GM volume will show a gradual decline with age, while WM volume will exhibit a nonlinear pattern, consistent with prior reports in other populations (4, 7–11).

## 2 Materials and method

### 2.1 Study design

This single-center, cross-sectional study was conducted at Bach Mai Hospital, one of the largest multidisciplinary hospitals in Vietnam, between October 2021 and September 2022. A total of 110 participants were enrolled. Ethical approval was obtained from the Institutional Review Board (IRB) of Hanoi Medical University, and all participants provided written informed consent before participation. The study adhered to the guidelines of the Strengthening the Reporting of Observational Studies in Epidemiology (STROBE) checklist, ensuring rigorous and transparent reporting (see Supplementary material).

### 2.2 Participants selection

Participants were enrolled in this study based on the following criteria:

1. Age within either the younger age group (18–35 years) or the older age group (60–80 years). The age selection was driven by resource constraints and prior literature suggesting that brain volume changes between ages 35 and 60 are relatively modest, with more pronounced atrophy typically occurring after age 60 (7, 11, 16). This design also aligns with the average life expectancy of the Vietnamese population, which is approximately 75 years (30);
2. Cognitively normal subjects (with MMSE score ≥ 24 points) (31);
3. No history of neuropsychiatric disorders (e.g., major depression, schizophrenia, epilepsy) or major chronic diseases known to affect brain structure, including cardiovascular diseases such as hypertension, diabetes mellitus, stroke, or prior head trauma, as confirmed during screening and medical record review;
4. Successful quantification of brain volume using the Quantib™ Brain tool;
5. Absence of abnormalities on morphological, vascular, and diffusion MRI;
6. And willingness to participate in the study as a volunteer.

Exclusion criteria were as follows: (1) presence of contraindications to MRI; (2) presence of any of the following clinical conditions: alcohol or substance addiction/ history of brain surgery or traumatic brain injury/ history of seizures, epilepsy, encephalitis, or meningitis/ diagnosed neurological or psychiatric disorders, or emotional disturbances; (3) evidence of pathological brain parenchymal abnormalities detected on conventional MRI (e.g., infarction, hemorrhage, tumor, or structural abnormalities), confirmed by a radiologist; or (4) inability to successfully measure brain volume using the Quantib™ Brain tool.

### 2.3 Participants screening

Volunteers for this study were recruited from individuals undergoing routine health check-ups at the Outpatient Department of Bach Mai Hospital. Initial screening was conducted by a neurologist to evaluate participants based on clinical inclusion and exclusion criteria. Subsequently, standard cerebral imaging assessments were performed by a radiologist to confirm eligibility prior to proceeding with Quantib™ Brain scans for brain volume measurements.

### 2.4 MRI acquisition

MRI acquisition was conducted on a 3 Tesla MRI system (GE Healthcare) using a standardized two-step protocol to ensure optimal image quality and diagnostic precision. The initial step involved MRI screening with standard sequences, including axial FLAIR, axial DWI, sagittal T1, and 3D TOF of the circle of Willis, to confirm normal brain morphology, evaluate vascular structures, and rule out acute or chronic pathological findings. Following radiological confirmation of normal findings, high-resolution 3D IR-prepared fast spoiled gradient-recalled acquisition (BRAVO sequence). Images were performed in the sagittal plan with the following parameters: TE = 3.8 ms, TR = 7.8 ms, TI = 600 ms, flip angle = 12°, FOV = 25 × 25 × 18 cm, slice thickness = 1 mm with no interslice gap, matrix = 252 × 227, and NEX = 1, resulting in an isotropic voxel size of 1 × 1 × 1 mm^3^. This meticulous two-step approach ensured both the exclusion of confounding pathological conditions and the acquisition of high-quality volumetric data for accurate analysis.

### 2.5 Measurement of brain volume parameters using Quantib™ brain tool

**Figure 1:**
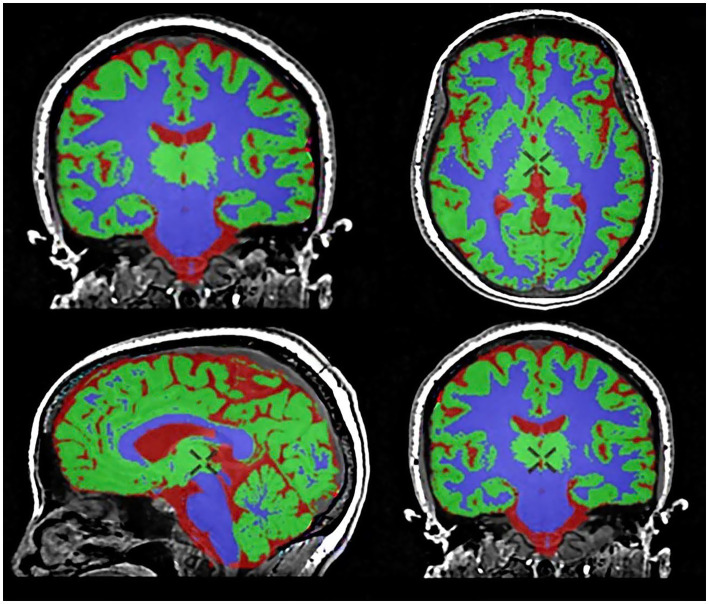
Brain segmentation and volumetric analysis using the Quantib™ Brain tool. Gray matter (GM) is segmented in green, white matter (WM) in violet, and cerebrospinal fluid in red.

The original 3D T1-weighted BRAVO images were seamlessly processed using the Quantib™ Brain tool integrated within the advanced GE Healthcare workstation. This tool provided an automated workflow for brain segmentation, volumetric quantification, visualization, and reporting (Figure 1) (29).

For each participant, the following brain volume indices were automatically measured and analyzed: (1) total gray matter volume (GMV), (2) total white matter volume (WMV), (3) total cerebrospinal fluid volume (CSF), and (4) total intracranial volume (TIV) = GMV + WMV + CSF. Furthermore, to account for interindividual differences in head size, normalized brain volumes were calculated: (5) normalized gray matter volume (nMV) = GMV / TIV, and (6) normalized white matter volume (nWM) = WMV / TIV. This standardized approach ensured robust and reproducible brain volume measurements, facilitating interparticipant comparisons while accounting for anatomical variability.

### 2.6 Statistical analysis

Continuous variables were expressed as mean ± standard deviation (SD), while categorical variables were presented as frequencies and percentages.

Comparisons of two mean values were conducted using the Student t-test for normally distributed data or the Mann–Whitney U test for non-normally distributed data. For comparisons involving multiple groups, analysis of variance (ANOVA) was applied for data with homogeneous variances and normal distribution, whereas the Kruskal-Wallis test was used for non-normally distributed data.

The relationships between volumetric brain parameters and age were evaluated using Pearson’s correlation coefficient for normally distributed data and Spearman’s rank correlation coefficient for non-normally distributed data. Correlation strengths were classified as follows: weak correlation with ∣r∣ < 0.30; moderate correlation with 0.30 ≤ ∣r∣ < 0.50; or strong correlation with ∣r∣ ≥ 0.70.

A *p*-value ≤ 0.05 was considered statistically significant for a 95% confidence interval (CI). All data analyses were performed using SPSS software (version 20.0; IBM SPSS Inc., Chicago, IL, United States).

## 3 Results

### 3.1 Participants characteristics

Between October 2021 and September 2022, a total of 110 consecutive participants were enrolled in this study. Of these, 108 participants (98.2%) successfully underwent brain volume assessment using the Quantib™ Brain tool without technical issues and were included in the final analysis. Two participants were excluded due to significant motion artifacts in the 3D T1-weighted BRAVO sequence, rendering their Quantib™ Brain results unreliable.

The distribution of participants across the four demographic groups was relatively balanced: 30 younger females (27.8%; mean age 29.6 ± 4.5 years), 27 younger males (25.0%; mean age 26.6 ± 4.8 years), 32 older females (29.6%; mean age 66.0 ± 3.9 years), and 19 older males (17.6%; mean age 65.8 ± 3.7 years). The mean brain volume indices for each group are detailed in Table 1.

**Table 1:** Average brain volume indices of the four subject groups (mean ± SD).

Brain Volume	Young female (*n* = 30)	Young male (*n* = 27)	Older female (*n* = 32)	Older male (*n* = 19)
GMV (cm^3^)	688.13 ± 50.58	767.19 ± 4.,97	62.28 ± 46.41	689.11 ± 52.96
WMV (cm^3^)	424.53 ± 33.04	475.22 ± 47.06	392.59 ± 30.17	458.47 ± 51.15
CSF (cm^3^)	229.77 ± 23.28	251.07 ± 34.41	257.28 ± 36.23	299.47 ± 38.30
TIV (cm^3^)	1342.43 ± 72.35	1493.48 ± 105.32	1279.16 ± 87.15	1447.05 ± 92.08
nGM	0.51 ± 0.02	0.51 ± 0.02	0.49 ± 0.02	0.48 ± 0.03
nWM	0.32 ± 0.02	0.32 ± 0.02	0.31 ± 0.02	0.32 ± 0.02

GMV, gray matter volume; WMV, white matter volume; CSF, cerebrospinal fluid, TIV, total intracranial volume; nGM, normalized gray matter; nWM, normalized white matter.

### 3.2 Differences in brain volume indices by age and sex

**Figure 2:**
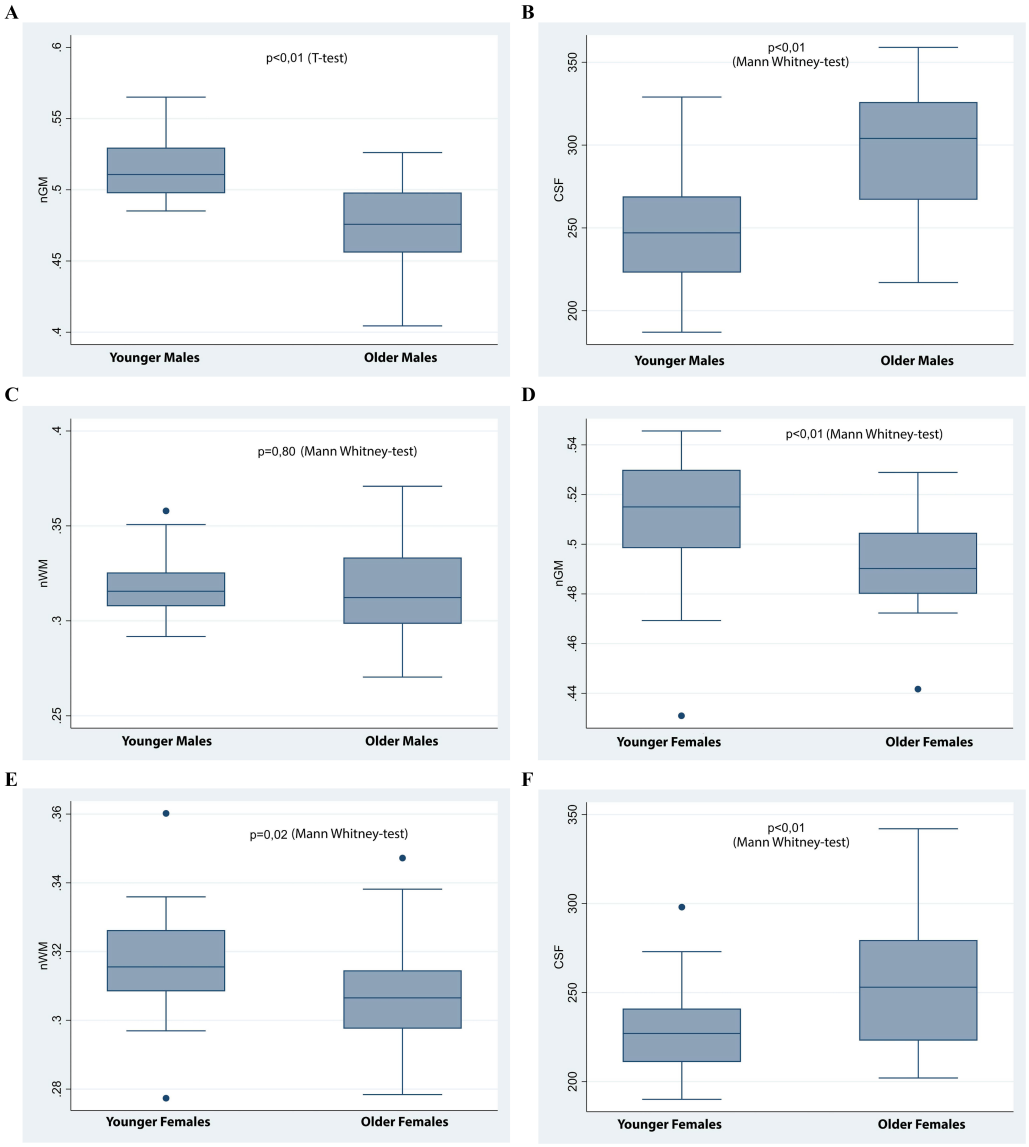
Box plots comparing brain volume indices across age and sex groups. **(A–C)** Comparisons between younger and older males. Significant differences were observed in normalized gray matter volume (nGM) **(A)** and CSF volume **(C)**, but not in normalized white matter volume (nWM) **(B)**. **(D–F)**: Comparisons between younger and older females. Significant differences were found in nGM **(D)**, nWM **(E)**, and CSF **(F)**.

Table 2 and Figure 2 highlights significant variations in brain volume indices influenced by age and sex. Younger participants exhibited significantly higher standardized gray matter volume (nGM) and standardized white matter volume (nWM) compared to older participants (*p* < 0.001 and *p* = 0.02, respectively), with no significant differences between sexes (*p* > 0.05). Cerebrospinal fluid (CSF) volume was significantly higher in males than females (*p* = 0.001) and in older participants compared to younger ones (*p* < 0.001). Total intracranial volume (TIV) was greater in males than females (*p* < 0.001) and in younger participants compared to older participants (*p* = 0.002).

**Table 2:** Age and sex related to brain volume indices.

Age-related	Young group (*n* = 57)	Old group (*n* = 51)	*p-*value
Normalized Gray Matter Volume (nGM)	0.51 ± 0.02	0.49 ± 0.02	<0.001^a*^
Normalized White Matter Volume (nWM)	0.32 ± 0.02	0.31 ± 0.02	0.02^a^
Cerebrospinal Fluid (CSF) Volume	239.86 ± 30.75	273.00 ± 42.03	<0.001^a*^
Total Intracranial Volume (TIV)	1413.98 ± 116.83	1341.71 ± 120.34	0.002^a*^

Sex-related	Female (*n* = 62)	Male (*n* = 46)	*p-*value
Normalized Gray Matter Volume (nGM)	0.502 ± 0.02	0.498 ± 0.03	0.51^a^
Normalized White Matter Volume (nWM)	0.31 ± 0.02	0.31 ± 0.02	0.10^a^
Cerebrospinal Fluid (CSF) Volume	243.97 ± 33.42	271.07 ± 43.03	0.001^a^*
Total Intracranial Volume (TIV)	1309.77 ± 85.81	1474.30 ± 101.66	< 0.001^a^*

a *p* was calculated by using the Student *t*-test.

*p < 0.05.

### 3.3 Correlation of brain volume indices with age and sex

**Figure 3:**
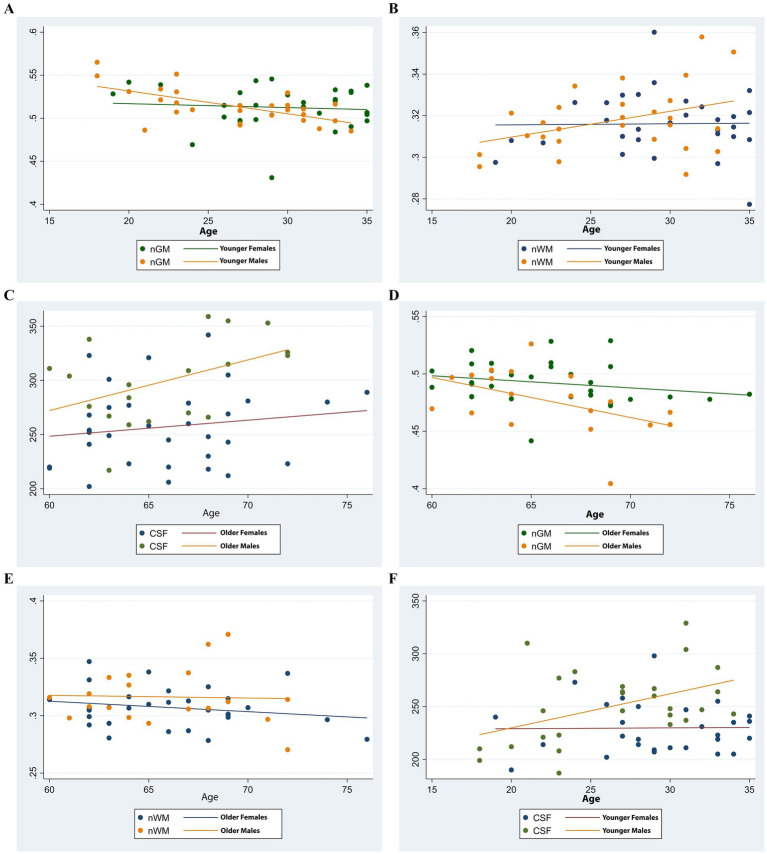
Scatter plots illustrating age-related trends in brain volume indices by sex. **(A,D)** Normalized gray matter volume (nGM) decreased with age in both younger and older groups, with a more pronounced decline observed in males. **(B,E)** Normalized white matter volume (nWM) showed a slight increase in younger males and remained relatively stable in younger females. In the older group, both sexes exhibited a decline, more markedly in females. **(C,F)** Cerebrospinal fluid (CSF) volume increased slightly in younger males while remaining stable in younger females. In the older group, CSF volume increased in both sexes, with a steeper trajectory in males.

This study identified significant correlations between brain volume indices and age, with variations based on sex. A negative correlation was observed between normalized gray matter volume (nGM) and age in younger males (r = −0.56, *p* < 0.001) and older males (r = −0.52, *p* = 0.02), whereas no significant correlation was found in females of any age group (*p* > 0.05). Normalized white matter volume (nWM) showed no significant correlation with age across any demographic group (*p* > 0.05). Cerebrospinal fluid (CSF) volume demonstrated a positive correlation with age in younger males (r = 0.41, *p* = 0.02) but not in other groups (*p* > 0.05). Total intracranial volume (TIV) did not correlate significantly with age in any group (*p* > 0.05). These findings, illustrated in Figure 3, highlight distinct patterns of brain volume changes in relation to age and sex.

## 4 Discussion

This prospective cross-sectional study explored age- and sex-related differences in gray matter volume (GMV), white matter volume (WMV), cerebrospinal fluid (CSF) volume, and total intracranial volume (TIV), in a cohort of cognitively normal Vietnamese adults. Using automated volume segmentation on high-resolution 3.0 Tesla MRI, our findings highlight significant age- and sex-related variations in brain volumes: (1) younger individuals exhibited significantly higher normalized gray matter volume (nGM) and white matter volume (nWM) compared to older individuals, with a notable inverse correlation between nGM and age observed in males but not females; (2) CSF volume was higher in older individuals and males, with a positive correlation with age observed only in young males; (3) TIV was significantly larger in males and younger individuals, with no significant correlation with age across all groups. These findings offer preliminary insights into the neuroanatomical characteristics of Vietnamese populations and contribute to the global understanding of brain volumetrics.

### 4.1 Age-related changes in brain volumes

Our results align with previous research reporting age-related reductions in GMV and WMV, coupled with increases in CSF volume, which reflect neurodegenerative processes associated with aging (32, 33). The significant decrease in nGM and nWM observed in older participants parallels findings from European and North American cohorts (34, 35). Additionally, studies on East Asian populations, including Japanese and Chinese cohorts, have reported similar age-related reductions in gray and white matter volumes, suggesting the universality of these patterns across diverse ethnic groups (36, 37). These reductions are thought to stem from cortical atrophy, neuronal loss, and myelin degeneration—hallmarks of aging (7, 16).

Interestingly, our correlation analysis revealed a significant inverse relationship between nGM and age in males, whereas no such correlation was observed in females. This finding is consistent with previous studies reporting greater age-related gray matter decline in males compared to females (38, 39). This sex-specific disparity may reflect hormonal influences, particularly the neuroprotective effects of estrogen in females (40–42). Further research is warranted to investigate the underlying mechanisms, including hormonal and genetic factors that might contribute to these differences.

### 4.2 Sex-related differences in brain volumes

Sex-related differences in brain volumetrics observed in our study partially align with existing literature (14–22). Focusing on Asian population, while no significant difference was found in nGM between males and females in our cohort (*p* = 0.51), a study by Farokhian et al. (43) on a Japanese cohort reported significantly higher nGM volumes in females. This discrepancy could be attributed to differences in population characteristics, emphasizing the importance of studying diverse cohorts, or to the smaller sample size in our study compared to theirs (108 vs. 277 participants).

Regarding nWM, our findings corroborate those of Farokhian et al., where younger participants had higher nWM than older participants, and males exhibited higher nWM than females (43). In contrast, males in our study had significantly larger CSF and TIV volumes than females, consistent with previous research (10, 43, 44). These sex-related differences likely reflect anatomical variations rather than pathological processes, underscoring the importance of considering demographic factors in volumetric analyses.

### 4.3 Correlation analysis

The inverse correlation between nGM and age in males but not females aligns with prior studies demonstrating sex-specific patterns in brain aging (45–47). For example, research indicates that while both sexes experience gray matter reductions with age, the trajectories and regional distributions of these changes differ significantly (43). Males generally exhibit earlier and more pronounced gray matter decline in frontal and temporal regions, whereas females tend to show more localized or delayed reductions, particularly after menopause (8, 16, 18). Several factors have been proposed to explain these divergent patterns, including hormonal influences – most notably the neuroprotective effects of estrogen in females – as well as genetic variations in susceptibility to neurodegeneration and differences in the rate of vascular aging between sexes (18, 48, 49).

Additionally, the positive correlation between CSF volume and age observed in young males, but not in other groups, is consistent with findings of greater CSF volume increases in men compared to women with aging (39, 47, 50, 51). This pattern may reflect differences in cortical atrophy rates and highlights the complex interplay of sex and age in brain aging. Longitudinal studies are needed to better understand these trajectories and their implications for neurodegenerative diseases.

### 4.4 Technique implications and novel contributions

This study represents the first prospective investigation of brain volumetrics in a Vietnamese cohort using high-field MRI and automated segmentation software (Quantib™ Brain). The low technical failure rate of Quantib™ Brain measurements in our study (1.8%) is encouraging and compares favorably to reports of higher failure rates due to software issues in other studies, such as the 9% failure rate reported by Mangesius et al. (52).

Our findings contribute to the limited body of literature on brain volumetrics in Southeast Asian populations, particularly in Mainland Southeast Asia, including Vietnam, Laos, and Cambodia. The lack of significant correlations between nWM and age in our cohort suggests potential regional or cohort-specific differences in white matter integrity, warranting further exploration.

The use of Quantib™ Brain software ensures high accuracy and reproducibility in volumetric analysis, reinforcing the reliability of our findings and providing a valuable reference for future research in the region.

### 4.5 Limitations

This study has several limitations. First, its cross-sectional design precludes causal inferences regarding age-related changes in brain volumes. Longitudinal studies are required to confirm these findings and elucidate the mechanisms underlying these changes. Second, the relatively small sample size, while sufficient for initial analysis, may limit the detection of subtle inter-individual differences. Third, the exclusion of individuals with neurological conditions restricts the applicability of our findings to clinical populations. Lastly, while Quantib™ Brain has been validated in multiple populations and is widely used in clinical practice, its performance in the Vietnamese population has not yet been specifically assessed. The absence of cross-validation with manual segmentation or other software tools such as FreeSurfer constitutes a limitation of this preliminary study and should be addressed in future research.

## 5 Conclusion

This preliminary study highlights age- and sex-related differences in brain volumetrics in a Vietnamese cohort, underscoring the importance of demographic factors in neuroimaging studies. Further research involving larger, more diverse, and representative samples is needed to validate and extend these findings.

## Data Availability

The original contributions presented in the study are included in the article/Supplementary material, further inquiries can be directed to the corresponding author.
