# Supplementary material for: Preliminary assessment of age- and sex-related differences in brain volume using Quantib™ brain quantification: a study in a Vietnamese cohort
Source: Front Neurol. 2025 Aug 22;16:1552559. doi: 10.3389/fneur.2025.1552559 (PMC12411775; doi:10.3389/fneur.2025.1552559)
Supplement: Supplementary file 1 [file Data_Sheet_1.pdf]

STROBE Statement—Checklist of items that should be included in reports of ***cross-sectional studies***

| | Item No | Recommendation |
|---|---|---|
| **Title and abstract** | 1 | (*a*) Indicate the study's design with a commonly used term in the title or the abstract<br>*Prospective cross-sectional study as stated in the Abstract on page 1 and Materials and Methods on page 2.* |
| | | (*b*) Provide in the abstract an informative and balanced summary of what was done and what was found<br>*Provided in Abstract on page 1.* |
| **Introduction** | | |
| Background/rationale | 2 | Explain the scientific background and rationale for the investigation being reported<br>*Included in the Introduction on page 2.* |
| Objectives | 3 | State specific objectives, including any prespecified hypotheses<br>*Included in the Introduction on page 2.* |
| **Methods** | | |
| Study design | 4 | Present key elements of study design early in the paper<br>*Included in the Materials and Methods on page 2.* |
| Setting | 5 | Describe the setting, locations, and relevant dates, including periods of recruitment, exposure, follow-up, and data collection<br>*Included in the Materials and Methods on pages 2, 3, and 4.* |
| Participants | 6 | (*a*) Give the eligibility criteria, and the sources and methods of selection of participants<br>*Included in the Materials and Methods on pages 2 and 3.* |
| Variables | 7 | Clearly define all outcomes, exposures, predictors, potential confounders, and effect modifiers. Give diagnostic criteria, if applicable<br>*Included in the Materials and Methods on pages 3 and 4.* |
| Data sources/ measurement | 8* | For each variable of interest, give sources of data and details of methods of assessment (measurement). Describe comparability of assessment methods if there is more than one group<br>*Included in the Materials and Methods on page 4.* |
| Bias | 9 | Describe any efforts to address potential sources of bias<br>*Addressed in the limitations paragraph in the Discussion on pages 7 and 8.* |
| Study size | 10 | Explain how the study size was arrived at<br>*Included in the Study design on page 2.* |
| Quantitative variables | 11 | Explain how quantitative variables were handled in the analyses. If applicable, describe which groupings were chosen and why<br>*Included in the Materials and Methods on page 4.* |
| Statistical methods | 12 | (*a*) Describe all statistical methods, including those used to control for confounding<br>*Included in the Materials and Methods on page 4.* |
| | | (*b*) Describe any methods used to examine subgroups and interactions<br>*Included in the Materials and Methods on page 4.* |
| | | (*c*) Explain how missing data were addressed<br>*Not applicable.* |
| | | (*d*) If applicable, describe analytical methods taking account of sampling strategy<br>*Not applicable.* |
| | | (*e*) Describe any sensitivity analyses<br>*Not applicable.* |

| | | |
|---|---|---|
| **Results** | | |
| Participants | 13* | (a) Report numbers of individuals at each stage of study—eg numbers potentially eligible, examined for eligibility, confirmed eligible, included in the study, completing follow-up, and analysed *Included in the Results on page 4.* |
| | | (b) Give reasons for non-participation at each stage *Included in the Results on page 4.* |
| | | (c) Consider use of a flow diagram *Not required.* |
| Descriptive data | 14* | (a) Give characteristics of study participants (eg demographic, clinical, social) and information on exposures and potential confounders *Included in the Results on page 4.* |
| | | (b) Indicate number of participants with missing data for each variable of interest *Not applicable.* |
| Outcome data | 15* | Report numbers of outcome events or summary measures *Included in the Results in Table 1.* |
| Main results | 16 | (*a*) Give unadjusted estimates and, if applicable, confounder-adjusted estimates and their precision (eg, 95% confidence interval). Make clear which confounders were adjusted for and why they were included *Included in the Results on page 2 and in Tables 1, 2.* |
| | | (*b*) Report category boundaries when continuous variables were categorized *Included in the Results on page 4 and in Tables 1, 2.* |
| | | (*c*) If relevant, consider translating estimates of relative risk into absolute risk for a meaningful time period *Not applicable.* |
| Other analyses | 17 | Report other analyses done—eg analyses of subgroups and interactions, and sensitivity analyses *Included in the Results on page 4 and in Figures 2, 3.* |
| **Discussion** | | |
| Key results | 18 | Summarise key results with reference to study objectives *Included in the Discussion on pages 4 and 5.* |
| Limitations | 19 | Discuss limitations of the study, taking into account sources of potential bias or imprecision. Discuss both direction and magnitude of any potential bias *Included in the Discussion on pages 7 and 8.* |
| Interpretation | 20 | Give a cautious overall interpretation of results considering objectives, limitations, multiplicity of analyses, results from similar studies, and other relevant evidence *Included in the Discussion on pages 5, 6, and 7.* |
| Generalisability | 21 | Discuss the generalisability (external validity) of the study results *Included in the Discussion on page 6.* |
| **Other information** | | |
| Funding | 22 | Give the source of funding and the role of the funders for the present study and, if applicable, for the original study on which the present article is based *Provided in the text on page 8.* |

*Give information separately for exposed and unexposed groups.
